# Extensive oral ulcer in a patient with lupus erythematosus

**DOI:** 10.5935/0103-507X.20190020

**Published:** 2019

**Authors:** Victor Hugo Patrocínio, Paulo Pereira Nascimento, Renata Lanzoni de Oliveira, Aline Joana Linhares Gurski Seco, Rejane Cristina Leite da Fonseca, Ellen Cristina Gaetti-Jardim

**Affiliations:** Universidade Federal do Mato Grosso do Sul - Campo Grande (MS), Brazil.

**To the editor,**

Systemic lupus erythematosus (SLE) is a heterogeneous and multisystemic autoimmune disease characterized by the production of autoantibodies against various cellular constituents.^(1)^ Systemic lupus erythematosus presents with varied clinical manifestations and periods of exacerbation and remission.^(2)^ Systemic lupus erythematosus may present as intraoral lesions that occur primarily in the tongue, buccal mucosa, clips and palate; chronic ulcers; or erythema of varying dimensions.^(3)^

A 38-year-old female patient with SLE reported increased volume in the right parotid region suggestive of viral parotitis and left otalgia, associated with a measured fever of 39°C. The patient also mentioned having taken antibiotics and analgesics (cephalexin and paracetamol) but observed no improvement, instead developing weakness and a decline in her general state. After seeking care at an urgent care unit, she was referred to hospital care and was admitted.

In the medical evaluation, a parotid ultrasound was requested five days after admission and showed evidence of preserved skin and subcutaneous tissue, parotid and submandibular glands of appropriate dimensions, preserved contours and echotexture, periglandular lymph nodes of usual appearance, and a lack of signs of nodules or collections. Therefore, significant changes were not found in the echographic exam.

**Figure 1:**
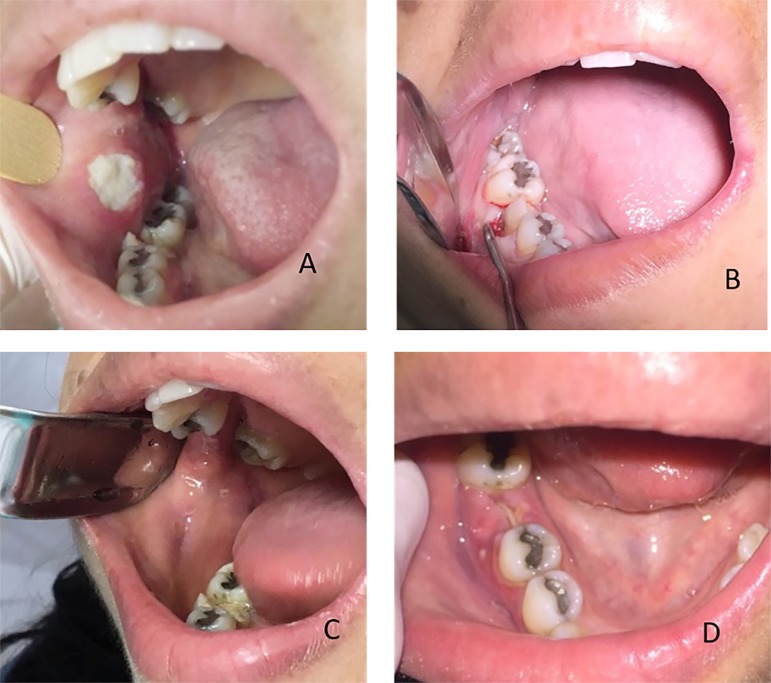
Clinical evaluation. (A) Initial clinical appearance. Extensive lesion on the jugal mucosa to the right of the defined limits in proximity to the first molar of the same side. (B) Tooth 46 with coronary fracture and sharp edges in proximity to the right jugal mucosa. (C) Clinical improvement of the jugal mucosa. Absence of ulcerated lesions. (D) Final clinical appearance after extraction of tooth 46.

Later, a dental evaluation was requested. Clinical examination at the bedside revealed a coronary fracture of element 46, a fistula in the region of the inserted gingival mucosa of the same dental element and a large ulcerated lesion in the jugal mucosa with regular contours and centers and high edematous borders, measuring approximately 3cm at the greatest extent (Figure 1A).

Due to the normality described in parotid ultrasonography, an abscess of odontogenic origin was suggested to have occurred, with the mucosal ulcer thought to be of traumatic origin due to a fractured dental crown. As an agreed-upon treatment among the team, the mucosal ulcer was initially treated with 0,1mg/mL, dexamethasone and a mouthwash of 0.12% chlorhexidine. Four days after the administration of the mouthwash, the lesion on the jugal mucosa was in remission. Subsequently, the subcutaneous dose of noxaparin 60mg enoxaparin every 12 hours was changed to 40mg 1x per day to prevent the risk of hemorrhage. Laboratory exams showed a hemoglobin level of 9.6g/dL, creatinine of 0.60mg dL, platelet counts of 305,000/mm^3^, International Normalized Ratio (INR) of 1,00, prothrombin time of activity (PTA) of 11.3 seconds, and activated partial thromboplastin time (aPTT) of 25.7 seconds. Element 46 was extracted in a hospital environment. Seven days after extraction, there was complete remission of the clinical picture, without symptomatic complaints (Figures 1B to 1D).

The extraction aimed for complete removal of the injured and infected tissue. Acute traumatic ulcers in the oral mucosa may present painful symptomatology, making feeding and hygiene difficult. The ulcers may clinically present as a lesion covered by off-white yellow exudates surrounded by a reddish halo. The dental surgeon must know the pathology to assist in the diagnosis of intraoral lesions and be able to differentiate it from other alterations that may be present in the oral cavity of SLE patients.^(4)^ It is important to know how to identify the origin of the trauma, which may be due to an orthodontic appliance, orotracheal tube, coronary fracture, or maladaptive prosthesis, among others. Once the traumatic agent has been identified, it should be removed so that there is no chronification of the lesion.^(5)^

*Victor Hugo Patrocínio* *Universidade Federal
do Mato Grosso do Sul - Campo Grande (MS), Brazil.* *Paulo Pereira Nascimento* *Universidade Federal do
Mato Grosso do Sul - Campo Grande (MS), Brazil.* *Renata Lanzoni de Oliveira* *Universidade Federal do
Mato Grosso do Sul - Campo Grande (MS), Brazil.* *Aline Joana Linhares Gurski Seco* *Universidade
Federal do Mato Grosso do Sul - Campo Grande (MS), Brazil.* *Rejane Cristina Leite da Fonseca* *Universidade
Federal do Mato Grosso do Sul - Campo Grande (MS), Brazil.* *Ellen Cristina Gaetti-Jardim* *Universidade Federal do
Mato Grosso do Sul - Campo Grande (MS), Brazil.*
